# Molecular serotyping and virulence assessment of *Avibacterium paragallinarum* strains circulating in Chinese poultry flocks

**DOI:** 10.3389/fvets.2026.1772264

**Published:** 2026-02-24

**Authors:** Jiaolong Wen, Zhangmin Liao, Lijuan Yin, Lin Zhu, Yanhua Xu, Xiaoling Wang, Gao Fan, Jianping Qin, Qingfeng Zhou, Wencheng Lin

**Affiliations:** 1 College of Veterinary Medicine & College of Animal Science, South China Agricultural University, Guangzhou, China; 2 Wen's Foodstuffs Group Co., Ltd., Yunfu, Guangdong, China; 3 Yantai Animal Disease Prevention and Control Center, Yantai, Shandong, China

**Keywords:** infectious coryza, *Avibacterium paragallinarum*, serotype, epidemiology, pathogenicity, China

## Abstract

**Background:**

Infectious coryza, caused by *Avibacterium paragallinarum* (*A. paragallinarum*), is a highly contagious respiratory disease that poses a significant threat to poultry production in China. Updated information on the epidemiology, serotype distribution, and virulence of circulating strains is essential for formulating effective regional control and vaccination strategies.

**Methods:**

A total of 200 clinical samples from nine provinces were analyzed to determine the prevalence, serotype distribution, and seasonal dynamics of *A. paragallinarum*. The bacterial isolation, characterization, species-specific PCR, and molecular serotyping based on the *hmtp210* gene were performed. Phylogenetic relationships were inferred using the neighbor-joining method. The pathogenicity of representative isolates (serotype A and C) was assessed using SPF chickens.

**Results:**

*A. paragallinarum* prevalence varied geographically, with the highest isolation rates in Anhui, Guangxi, and Guangdong provinces, and no detection in Sichuan or Hubei. A distinct seasonal pattern was observed, with peaks in late autumn and winter. 22 isolates were recovered and serotyped: serotype A predominated (*n* = 15), followed by serotype C (*n* = 5), and serotype B (*n* = 2). Phylogenetic analysis of *hmtp210* revealed clear clustering congruent with serotype classification. Both serotypes induced typical infectious coryza, with directly inoculated birds exhibiting more severe clinical signs than contacts.

**Conclusion:**

This multi-provincial study provides contemporary insights into the epidemiology, serotype distribution, and virulence of *A. paragallinarum* in China. These findings underscore the necessity for region-specific surveillance and support the development of targeted vaccines based on locally prevalent, virulent strains.

## 1 Introduction

Infectious coryza (IC) is an acute, highly contagious respiratory disease of chickens that poses a significant threat to global poultry production. The causative agent, *Avibacterium paragallinarum* (*A. paragallinarum*), is a Gram-negative, capsulated, nicotinamide adenine dinucleotide (NAD)-dependent bacterium of the family *Pasteurellaceae* (1, 2). Clinically, the disease is characterized by sneezing, facial swelling, lacrimation, conjunctivitis, and serous-to-mucopurulent nasal discharge (1, 3, 4). While chickens of all ages are susceptible, the economic impact is most pronounced in commercial settings (1, 5). Broilers suffer from reduced feed intake and growth retardation, while layers can experience egg production drops of up to 40% (1). Environmental stressors and concurrent infections often exacerbate clinical signs and severity, contributing to widespread flock outbreaks (6, 7).

Since its initial description in 1931, *A. paragallinarum* has been identified worldwide (1, 8). In China, Infectious coryza was first documented in Beijing and subsequently detected across numerous provinces, including Jiangsu, Hebei, Anhui, and Ningxia (4). The capsular polysaccharide is a primary virulence factor and the key protective antigen, making it a major focus for vaccine development (9, 10). The classical Page serotyping scheme based on capsular antigenicity classifies *A. paragallinarum* into three serovars (A, B, and C) without cross-protect (11). This scheme was later refined by Kume, who defined serogroups I, II, and III as equivalents to Page’s serovars A, B, and C, respectively (12). To date, nine recognized serovars have been identified (A-1 to A-4, B-1, and C-1 to C-4) (12, 13).

Levels of cross-protection within serovars vary considerably. Serovar A strains generally induce strong mutual protection, serovar B provides only partial protection, and serovar C offers minimal cross-protection. Furthermore, some field isolates remain untypable (1, 14). All three major serovars have been identified in China, with recent epidemiological data revealing an increasing incidence of IC. The current landscape is characterized by the predominance of serovars A and C, alongside the occasional co-circulation of all three types (3, 8, 15).

Accurate serotyping is essential for epidemiological investigation and vaccine selection. However, conventional haemagglutination-inhibition (HI) assays, which rely on high-quality standard antisera, are hampered by challenges in reagent availability and standardization, often yielding ambiguous results (14). To overcome these limitations, molecular serotyping based on sequence polymorphisms in the *hmtp210* gene has been established as a reliable alternative. Previous reports indicated that variability within the N-terminal 1–400 amino acid region of hmtp210 protein accurately differentiates Page serovars A, B, and C (14). The discriminatory power was further enhanced by Li et al. (16), who showed that analysis of both the 1–400 and 1,100–1,600 regions provide superior resolution. Consequently, sequencing of *hmtp210* gene has become the molecular gold standard for serotyping *A. paragallinarum*.

Given the substantial economic impact of IC in China and the dynamic, regionally variable distribution of *A. paragallinarum* serovars, continuous and targeted epidemiological surveillance is crucial. This study aimed to characterize the contemporary epidemiological profile of *A. paragallinarum* in China. Clinical samples were screened for *A. paragallinarum*, and confirmed by species-specific PCR, and subsequently molecularly serotyped based on *hmtp210* gene sequencing. Furthermore, the pathogenicity of representative strains from the predominant serotypes was assessed. The findings provide updated data on the geographical distribution, molecular characteristics, and virulence of circulating strains. These insights are critical for informing evidence-based prevention strategies and guiding the development of effective, regionally tailored vaccines.

## 2 Materials and methods

### 2.1 Ethics statement

All animal procedures were reviewed and approved by the Animal Care Committee of South China Agricultural University (approval ID: SYXK-2024-0136). All procedures complied with the Guidelines for the Care and Use of Laboratory Animals issued by the Ministry of Science and Technology of China. To minimize distress, birds were euthanized following deep isoflurane anesthesia and subsequent intravenous pentobarbitone administration, according to established broiler anesthesia protocols (17).

### 2.2 Sample collection

A systematic epidemiological investigation of IC was conducted from September 2024 to October 2025 across nine provinces in China (Guangdong, Guangxi, Jiangsu, Zhejiang, Sichuan, Hubei, Hebei, Henan, and Anhui). Swabs from the infraorbital sinuses and/or nasal exudates were collected from chickens exhibiting characteristic clinical signs of IC, including facial swelling and nasal discharge. Samples were transported under cold-chain conditions and refrigerated at 4 °C until processing. A total of 200 samples were obtained, and their geographic distribution is detailed in Table 1. The number of samples obtained from each province was determined by the availability of clinically suspected IC cases and voluntary diagnostic submissions during the study period, rather than by a predefined or evenly stratified sampling design. Consequently, sample sizes varied substantially among provinces.

**Table 1:** Prevalence of *A. paragallinarum* in this study.

Province	Apg positive rate	Serotype A	Serotype B	Serotype C
Guangdong	73.33% (11/15)	8	1	2
Guangxi	75.81% (47/62)	24	1	22
Zhejiang	30.77% (4/13)	0	0	4
Jiangsu	65.82% (52/79)	29	0	23
Sichuan	0% (0/7)	0	0	0
Hubei	0% (0/6)	0	0	0
Hebei	63.64% (7/11)	7	0	0
Anhui	100% (4/4)	4	0	0
Henan	66.67% (2/3)	2	0	0

Apg = *Avibacterium paragallinarum*.

### 2.3 Isolation and identification of *Avibacterium paragallinarum*

Following surface disinfection of the head, infraorbital sinuses were aseptically incised. Exudates were collected using sterile inoculating loops and streaked onto tryptic soy agar (TSA) supplemented with 10% heat-inactivated chicken serum and 0.02% *β*-nicotinamide adenine dinucleotide (β-NAD). Plates were incubated at 37 °C in a 10% CO_2_ atmosphere for 24 h. Presumptive colonies exhibiting smooth, dew-drop-like morphology were subcultured in tryptic soy broth (TSB) containing 10% chicken serum and 0.01% NAD, and incubated at 37 °C for 6 h with shaking (180 rpm). For purification, 3–5 single colonies were streaked to obtain pure cultures. Colony and cellular morphology were examined visually and via Gram staining and microscopy, respectively. Species-level identification was confirmed by PCR amplification of an ~1,600 bp fragment from the *hmtp210* gene using established primers (18). Isolates yielding the expected amplicon were identified as *A. paragallinarum*. A previously characterized *A. paragallinarum* reference strain (CVCC4341) was included as a positive control in each PCR run to verify assay specificity and performance, and its identity had been confirmed by sequencing. Due to limitations related to sample quality, transport conditions, and biosafety requirements, bacterial isolation was attempted only for a subset of PCR-positive samples with sufficient volume and appropriate storage conditions.

### 2.4 PCR amplification and phylogenetic analysis

Bacterial genomic DNA was extracted exclusively from purified *A. paragallinarum* colonies obtained as described in this study. DNA extraction was conducted using the FastPure Microbiome DNA Isolation Kit (Cat. no. DC502; Vazyme Biotech, Nanjing, China) according to the manufacturer’s instructions (Version 24.1). Briefly, approximately 400 μL of bacterial culture was transferred to a lysis tube and centrifuged at 10,000 rpm for 3 min to pellet the cells. After supernatant removal, the pellet was resuspended and incubated at 70 °C for 5 min to ensure efficient cell lysis. Following centrifugation (10,000 rpm, 1 min), the lysate was mixed with absolute ethanol and loaded onto a silica-based DNA binding column. The column was washed sequentially with Buffer WP and Buffer WB, dried by high-speed centrifugation, and genomic DNA was eluted in 50 μL of elution buffer. DNA concentration and purity were assessed using a NanoDrop 2000 spectrophotometer (Thermo Fisher Scientific, USA), and extracted DNA was stored at −20 °C until further use.

The *hmtp210* gene was amplified using specific primers: HMTp210F (5’-ATGAATAAAGTTTTTAAAATTAAATATTCTGTTATTAAACAAGAAAT-3′) and HMTp210R (5’-CTAAAAGGTAAATCCAACACTCATTGCTGCCC-3′), which generate an approximately 6100 bp amplicon (19). PCR reactions (50 μL) consisted of 25 μL PrimeSTAR HS Premix (Takara Bio), 10 pM of each primer, and 2 μL of template DNA. Reactions were carried out on a T100 Thermal Cycler (Bio-Rad Laboratories, Hercules, CA, United States) under the following cycling conditions: an initial denaturation at 98 °C for 3 min; 32 cycles of denaturation at 98 °C for 15 s, annealing at 55 °C for 10 s, and extension at 72 °C for 35 s; followed by a final extension at 72 °C for 5 min. PCR products were held at 4 °C until further analysis. Amplified PCR products were separated by 1% agarose gel electrophoresis. Target bands were excised and purified using a gel extraction kit (Solarbio). Purified amplicons were A-tailed using a DNA A-Tailing Kit (Takara Bio), ligated into the pMD19-T vector (Takara Bio), and transformed into *Escherichia coli DH5α* competent cells. For each isolate, three independent positive clones were selected for bidirectional sequencing by Sangon Biotech (Shanghai, China). Sequences were assembled and analyzed using the SeqMan program of DNASTAR Lasergene 7.1 software. The *hmtp210* sequences from the 22 obtained isolates were aligned with those from 15 reference strains (Table 2) using the ClustalW algorithm in MEGA11. A phylogenetic tree was constructed via the neighbor-joining method with 1,000 bootstrap replicates to assess genetic relationships and molecular serotype classification.

**Table 2:** *Avibacterium paragallinarum* reference strains retrieved from the GenBank database and clinical isolates generated in this study.

Apg strain	Accession no.	Country	Serotype	Year	Note
221	KJ867495.1	Japan	A	Unknown	Reference strains
13606.2/2009	PQ049067.1	USA	A	2009	
8	PQ804752.1	China	A	2011	
CVCC4341	PQ804769.1	China	A	2020	
2,403	PQ049057.1	USA	A	2024	
E-3C	PQ049059.1	Brazil	A	Unknown	
HP-14	PQ049060.1	USA	A	2024	
Spross	MW533118.1	Unknown	B	Unknown	
0222	PQ048980.1	USA	B	2024	
strain H18	KJ867496.1	Japan	C	Unknown	
TW94	KJ867497.1	Taiwan	C	1994	
Modesto	PQ049029.1	USA	C	1999	
TW07	KJ867498.1	Taiwan	C	2007	
TW13	MT050506.1	Taiwan	C	2013	
HP60	PQ049031.1	USA	C	2024	
Apg-21-GD	PX735107	China	A	2024	This study isolates
Apg-39-HB	PX735108	China	A	2024	
Apg-51-AH	PX735109	China	A	2024	
Apg-82-GX	PX735110	China	C	2024	
Apg-100-GD	PX735111	China	A	2024	
Apg-108-GX	PX735112	China	C	2024	
Apg-114-HB	PX735113	China	A	2024	
Apg-117-JS	PX735114	China	A	2025	
Apg-118-JS	PX735115	China	A	2025	
Apg-122-JS	PX735116	China	C	2025	
Apg-127-GX	PX735117	China	A	2025	
Apg-129-JS	PX735118	China	C	2025	
Apg-130-JS	PX735119	China	C	2025	
Apg-132-GX	PX735120	China	A	2025	
Apg-138-GD	PX735121	China	B	2025	
Apg-140-JS	PX735122	China	A	2025	
Apg-150-GX	PX735123	China	A	2025	
Apg-152-GX	PX735124	China	A	2025	
Apg-159-GX	PX735125	China	A	2025	
Apg-170-JS	PX735126	China	A	2025	
Apg-173-JS	PX735127	China	A	2025	
Apg-186-GX	PX735128	China	B	2025	

### 2.5 Animal experiment

The serotype A isolate Apg-159-GX and the serotype C isolate Apg-108-GX used in this study were clinical isolates obtained in the present investigation and were selected from the 22 confirmed *Avibacterium paragallinarum* isolates for challenge experiments. To assess the pathogenicity and transmissibility, an experimental infection study was performed using 50-day-old specific pathogen-free (SPF) chickens. Birds were randomly assigned into three groups: Group 1 (*n* = 15) was inoculated with serotype A isolate Apg-159-GX, Group 2 (*n* = 15) with serotype C isolate Apg-108-GX, and Group 3 (*n* = 15) served as the unchallenged control. For Groups 1 and 2, ten birds per group were directly challenged via inoculation into the left infraorbital sinus with 0.2 mL of a bacterial suspension containing 1 × 10^4^ CFU/mL. The remaining five birds per group were housed as in-contact sentinels to assess horizontal transmission. Control group birds received 0.2 mL of phosphate-buffered saline (PBS) via the same route. The challenge dose was selected based on previously published experimental infection models of infectious coryza, in which an inoculum of approximately 10^4^ CFU was demonstrated to reliably induce typical clinical signs without causing excessive mortality (20).

All chickens were monitored daily for 8 days post-challenge (dpc). Clinical signs were evaluated using a standardized clinical scoring system adapted from a previously published method for infectious coryza, with minor modifications to better reflect the severity of infraorbital sinus involvement observed under the present experimental conditions (8). Briefly, clinical signs were scored as follows: 0 = no signs; 1 = mild facial swelling/serous exudate; 2 = moderate swelling with mucous nasal discharge; 3 = severe swelling with copious exudate and/or transient blindness. At 8 dpc, all surviving birds were humanely euthanized. Necropsy was performed, with a specific focus on the infraorbital sinuses to confirm the presence and severity of caseous exudate characteristic of infectious coryza.

### 2.6 Statistical analysis

Statistical analyses were conducted using GraphPad Prism 9 (GraphPad Software Inc., San Diego, United States). Differences in clinical scores between groups across time points were analyzed using two-way analysis of variance (ANOVA). A *p*-value < 0.05 was considered statistically significant.

## 3 Results

### 3.1 Spatiotemporal distribution and serotype diversity of *Avibacterium paragallinarum*

**Figure 1:**
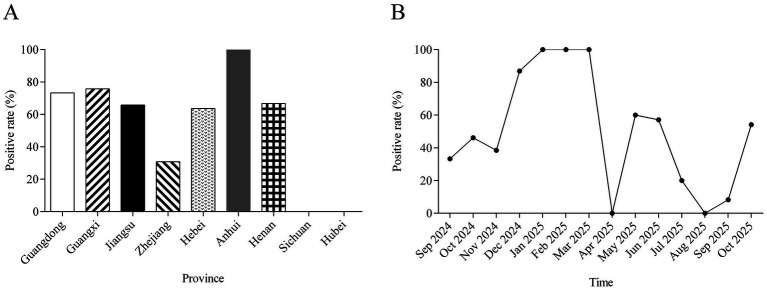
Spatiotemporal distribution of *A. paragallinarum* in China. **(A)** Geographic distribution of *A. paragallinarum* positivity across nine provinces. **(B)** Monthly positivity rates of *A. paragallinarum*.

A total of 200 clinical samples collected from nine major poultry-producing provinces were analyzed to determine the geographic distribution, seasonal patterns, and serotype composition of *A. paragallinarum*. *A. paragallinarum* detection varied substantially among provinces (Figure 1A; Table 1). The highest positivity rates were observed in Anhui (100.0%, 4/4), followed by Guangxi (75.8%, 47/62) and Guangdong (73.3%, 11/15). Moderate detection rates were recorded in Jiangsu (65.8%, 52/79), Henan (66.7%, 2/3), and Hebei (63.6%, 7/11), while a relatively low rate was found in Zhejiang (30.8%, 4/13). All samples from Sichuan (0/7) and Hubei (0/6) tested negative, revealing a pronounced regional heterogeneity with circulation concentrated primarily in southern and eastern China. These provincial detection rates should be interpreted with caution, particularly for regions with limited sample numbers, as the present study was not designed to estimate province-level prevalence.

Serotyping identified clear geographic differences in serotype distribution (Table 1). Serotype A was the most widespread, detected in Jiangsu (29 positive samples), Guangxi (24 positive samples), Guangdong (8 positive samples), Hebei (7 positive samples), Anhui (4 positive samples), and Henan (2 positive samples). In contrast, serotype B was identified in only one sample from Guangxi and one from Guangdong. Serotype C exhibited regional clustering, predominating in Jiangsu (23 positive samples) and Guangxi (22 positive samples), with lower occurrence in Zhejiang (4 positive samples) and Guangdong (2 positive samples). Serotype C was absent from Sichuan, Hubei, Hebei, Anhui, Henan. These results indicate co-circulation of serotypes A and C in key provinces (e.g., Jiangsu, Guangxi, Guangdong), a more restricted distribution of serotype A in northern/central regions, and an apparent absence of serotype B during the study period.

Seasonal dynamics revealed that a clear seasonal pattern was observed (Figure 1B). The positive rate increased from 33.3% in September 2024 to a peak of 87.0% in December 2024. A sustained high-incidence phase occurred from January to March 2025 (100.0%), followed by a sharp decline to 0% in April 2025. Positivity remained low throughout May–September. A distinct resurgence was observed in October 2025 (54.2%), establishing a stable, annual epidemic cycle with peak activity during late autumn and winter.

### 3.2 Isolation and identification of *Avibacterium paragallinarum*

**Figure 2:**
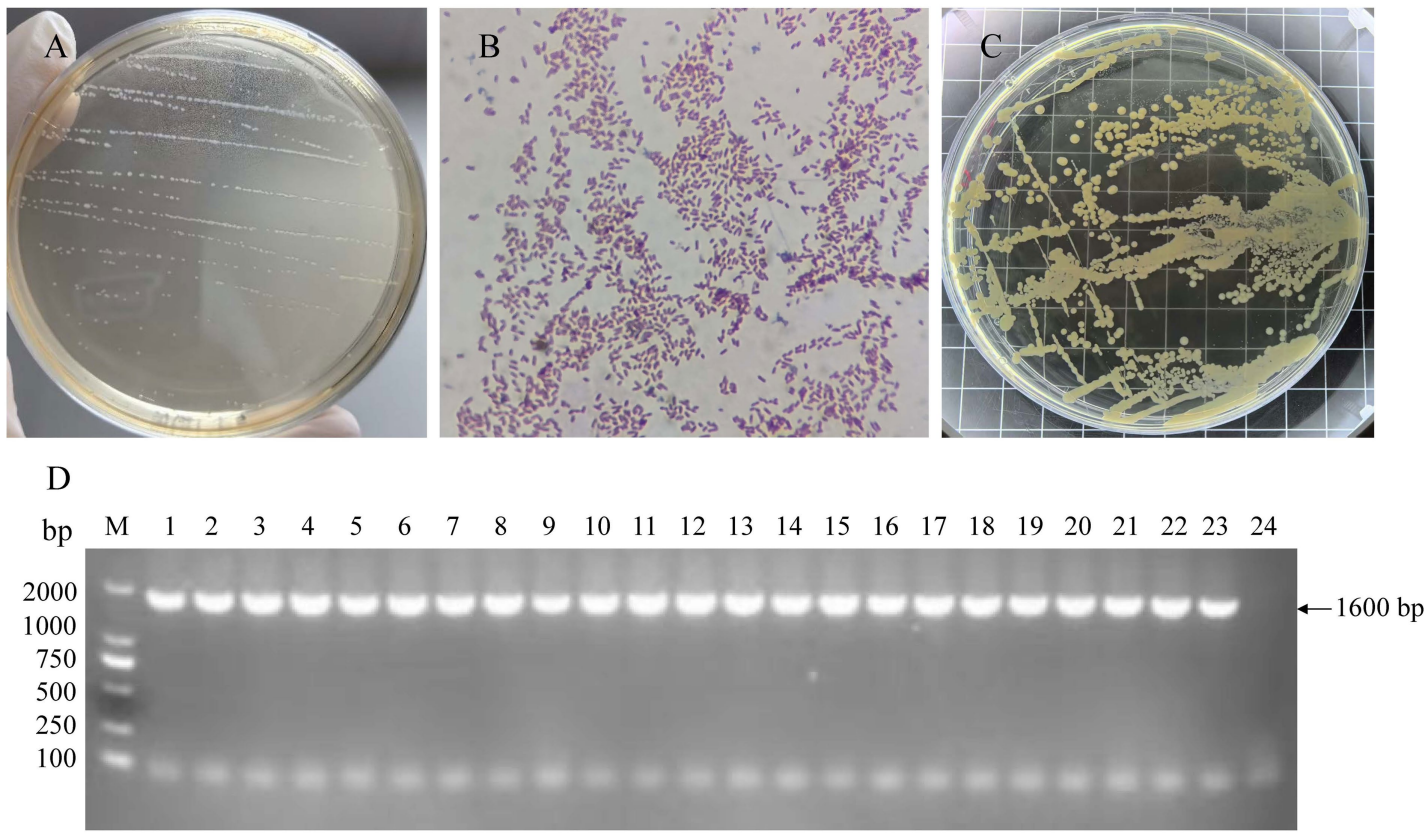
Isolation and characterization of *A. paragallinarum* field isolates. **(A)** Characteristic grey-white, dew-drop-like colonies of *A. paragallinarum* on supplemented TSA. **(B)** Gram-negative coccobacilli morphology of *A. paragallinarum* isolates. **(C)** Satellite growth around *Staphylococcus aureus*. **(D)** PCR amplification of the *hmtp210* gene. M, DL2000 DNA marker; lanes 1–22 correspond to PCR-positive clinical isolates confirmed in this study, and lane 23 represents the positive control (*A. paragallinarum* reference strain [CVCC4341]); lane 24, negative control.

*A. paragallinarum* was isolated from infraorbital sinus exudates collected from clinically suspected IC cases. From the 200 field samples, 22 isolates with phenotypic characteristics consistent with *A. paragallinarum* were recovered. Although a substantially larger number of samples tested positive by PCR, successful bacterial isolation was limited to these 22 isolates. On TSA supplemented with 10% chicken serum and NAD, colonies appeared as grey-white, round, smooth, and dew-drop-like (Figure 2A). Microscopic examination after Gram staining confirmed Gram-negative coccobacilli (Figure 2B). All isolates exhibited the characteristic satellite growth around *Staphylococcus aureus* streaks, confirming their NAD dependency (Figure 2C). To ensure clonal purity, isolates were subcultured, and 3–5 well-isolated colonies from each were subjected to species-specific PCR targeting the *hmtp210* gene. All selected colonies yielded the expected ~1,600 bp amplicon (Figure 2D), confirming their identity as *A. paragallinarum* and verifying the successful isolation of genetically representative strains.

### 3.3 Phylogenetic analysis of the *hmtp210* gene

**Figure 3:**
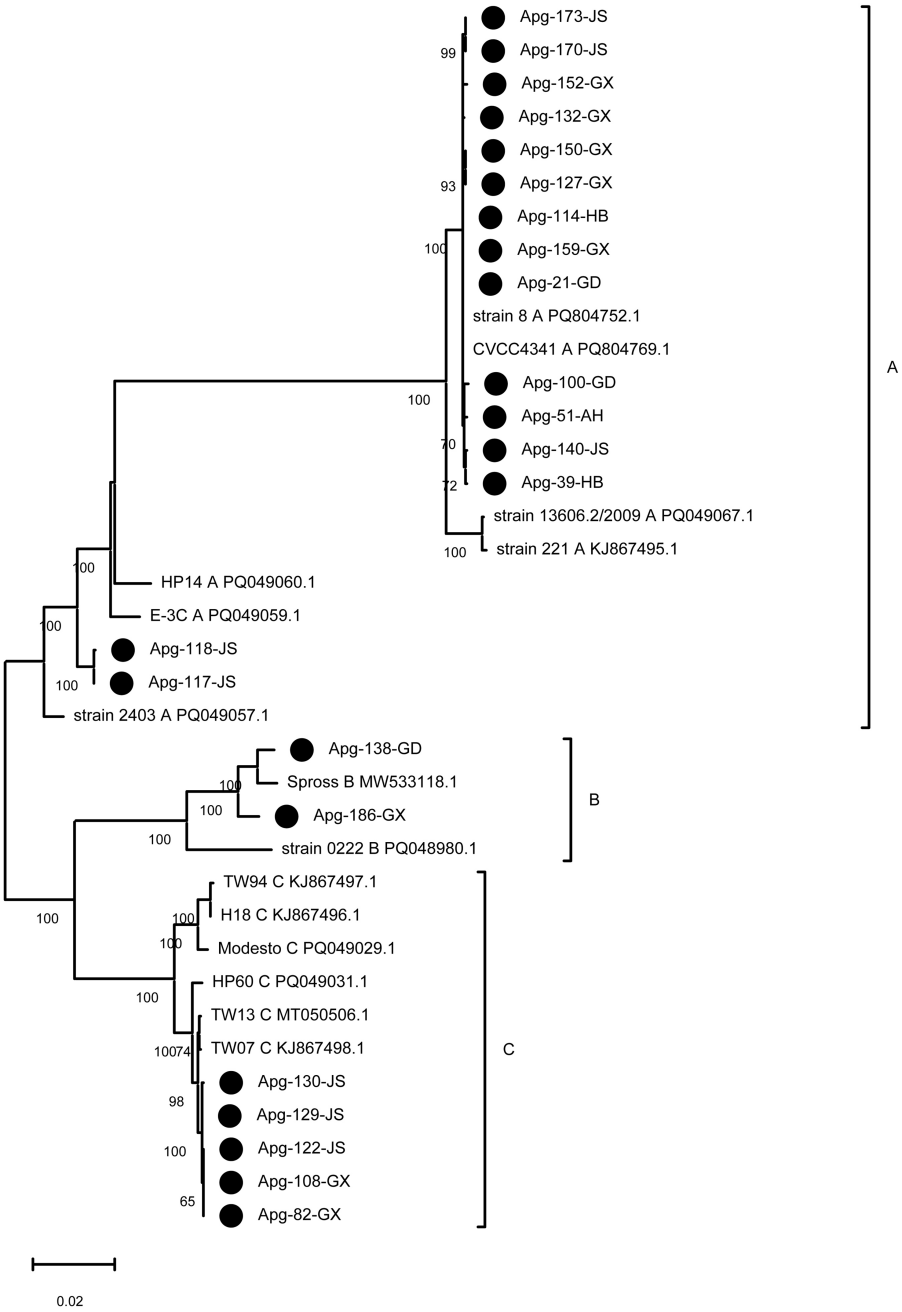
Phylogenetic analysis based on the *hmtp210* gene sequences. The phylogenetic tree was constructed using MEGA 7.0 based on *hmtp210* sequences from 22 isolates obtained in this study and 15 reference strains. Black circles denote the filed strain isolated in this study. Bootstrap values were calculated with 1,000 replicates.

A phylogenetic tree was constructed based on the *hmtp210* gene sequences from the 22 isolates and 15 reference strains representing serotypes A, B, and C. The GenBank accession numbers of the clinical isolates and reference strains are listed separately in Table 2. The resulting phylogeny (Figure 3) revealed that all isolates clustered into three distinct, well-supported clades corresponding to serotypes A, B and C, demonstrating strong concordance between molecular genotyping and conventional serotyping.

The majority of isolates (15 out of 22) formed a monophyletic clade with 7 reference serotype A strains, indicating a genetically homogeneous lineage circulating in the sampled regions. Two isolates clustered closely with a reference serotype B strain Spross. The remaining 5 isolates clustered with 2 reference serotype C strains (TW07, TW13), reflecting their close evolutionary relationship. These results confirm the predominance of serotype A among circulating strains in the sampled regions during the study period.

### 3.4 Clinical manifestations and pathogenicity of *Avibacterium paragallinarum*

**Figure 4:**
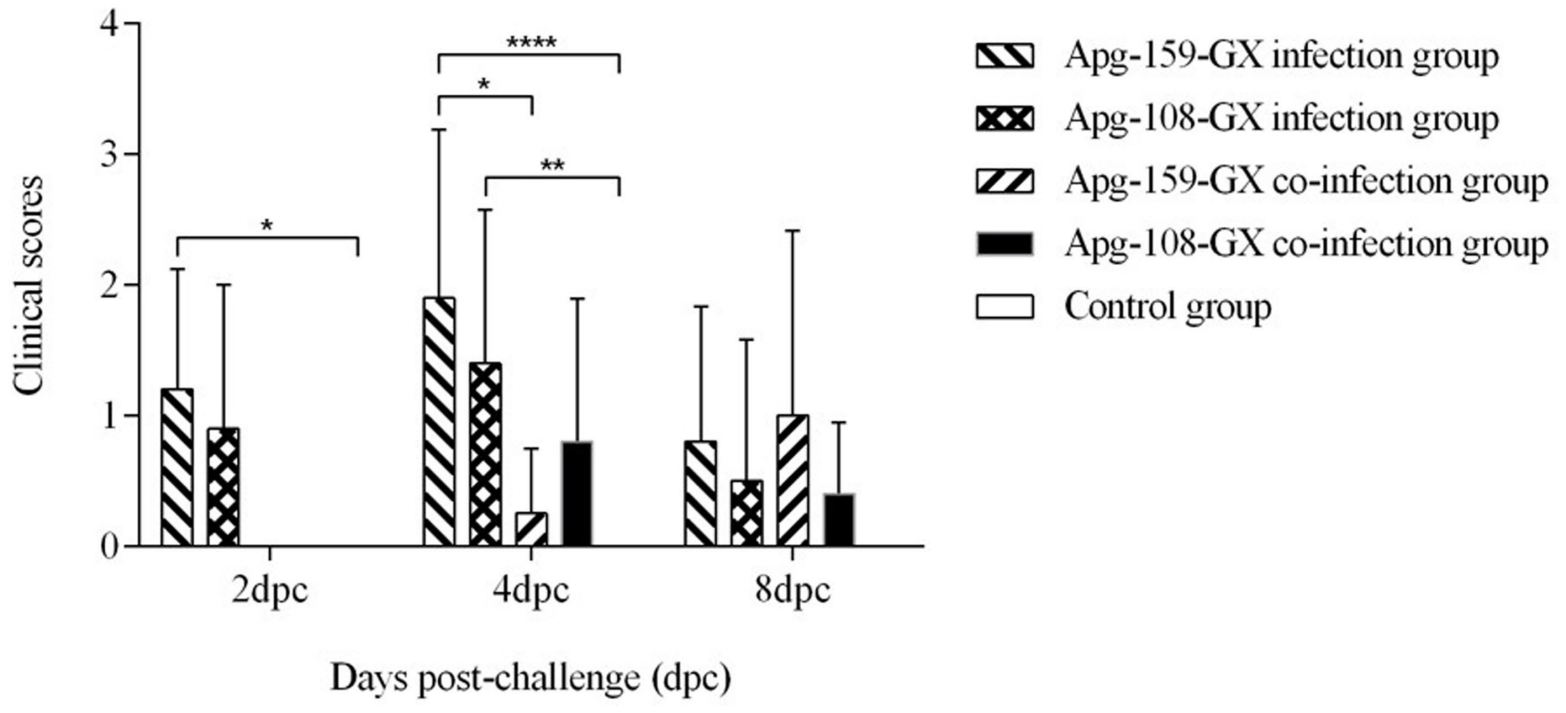
Clinical severity following experimental infection. Chickens were challenged with serotype A strain APG-159-GX or serotype C strain APG-108-GX via direct inoculation or contact exposure. Clinical signs were scored at 2, 4, and 8 days post-challenge (dpc).

Among the 22 confirmed *A. paragallinarum* isolates, Apg-159-GX (serotype A) and Apg-108-GX (serotype C) were selected for experimental challenge. Following direct infraorbital sinus inoculation of SPF chickens, both representative strains—Apg-159-GX (serotype A) and Apg-108-GX (serotype C)—induced typical infectious coryza. Clinical signs emerged at 2 dpc, peaked at 4 dpc, and largely resolved by 8 dpc. As shown in Figure 4, directly inoculated birds from both serotype groups exhibited significantly higher clinical scores than controls at 2 and 4 dpc (*p* < 0.05). At 4 dpc, clinical scores in the Apg-159-GX direct-challenge group were also significantly higher than in its contact-exposed group (*p* < 0.05), whereas no significant difference was observed between the Apg-108-GX challenge and contact groups (*p* > 0.05). By 8 dpc, clinical scores did not differ significantly among any groups (*p* > 0.05). No significant differences in mean clinical scores were observed between the two serotypes under direct-challenge conditions, indicating comparable overall clinical virulence in this model.

**Figure 5:**
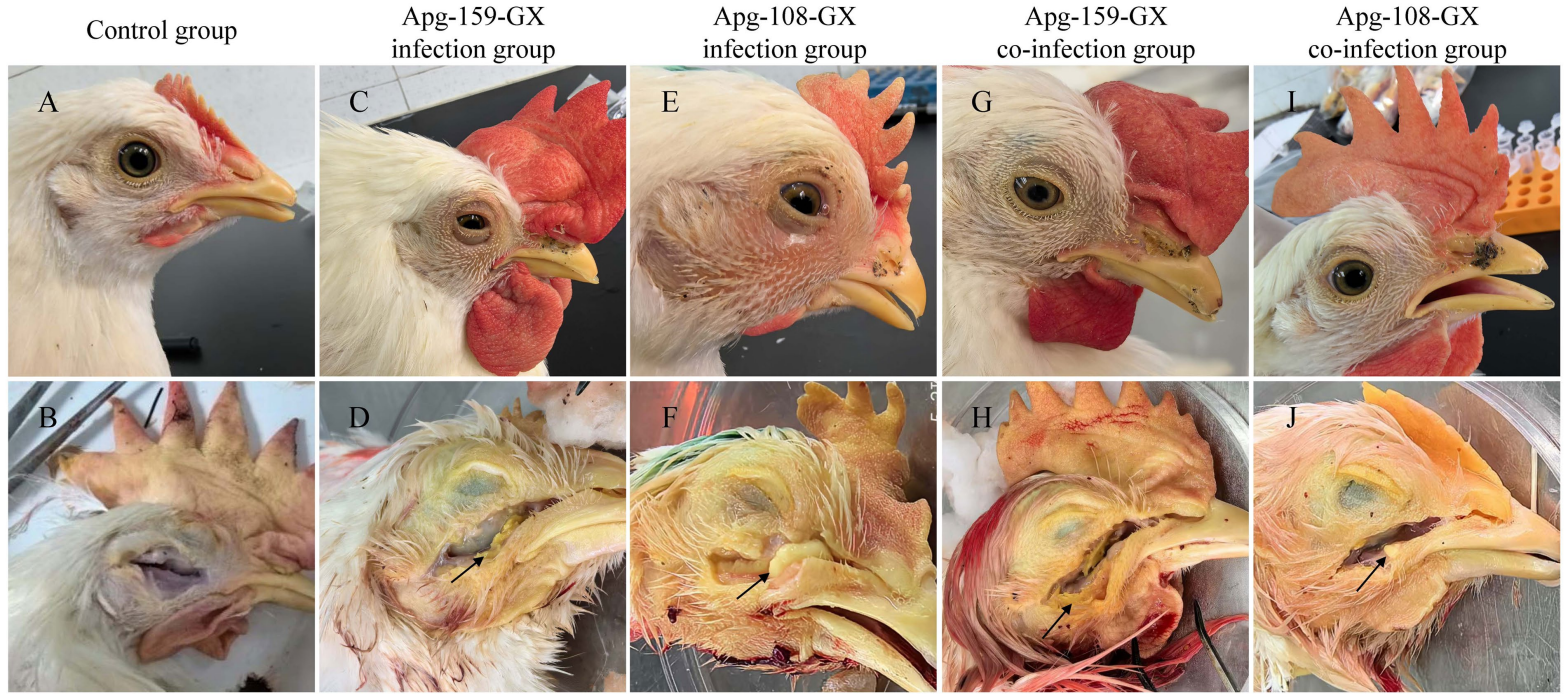
Gross pathology of SPF chickens infected with *A. paragallinarum*. Control chickens without clinical signs **(A)** and gross lesions in the infraorbital sinus **(B)**. **(C,D)** Serotype A strain Apg-159-GX—partially closed eyes with conjunctivitis and moderate periocular swelling **(C)**, and accumulation of yellow caseous exudate in the sinus **(D)**. **(E,F)** Serotype C strain Apg-108-GX—nasal discharge, lacrimation, and moderate swelling **(E)**, with the sinus filled with yellow caseous material **(F)**. **(G,H)** Contact-exposed birds with small amounts of caseous exudate in Apg-159-GX inoculated group **(H)**. **(I,J)** Contact-exposed birds with small amounts of caseous exudate in Apg-108-GX group.

Gross pathology. Gross lesion assessment corroborated the clinical findings (Figure 5). Control birds showed no abnormalities (Figures 5A,B). Directly challenged birds from both serotype groups developed classic IC lesions, including periocular swelling, conjunctivitis, and accumulation of yellow caseous exudate in the infraorbital sinus (Figures 5C–F). Contact-exposed birds displayed similar but milder lesions (Figures 5G–J), consistent with a lower infectious dose during natural transmission.

## 4 Discussion

*A. paragallinarum*, the etiological agent of infectious coryza (IC), has re-emerged repeatedly in China over the past decade, imposing significant economic losses on the poultry industry. IC is a highly contagious respiratory disease characterized by conjunctivitis, sinusitis, nasal discharge, facial swelling, and depression (1). Beyond causing reduced feed intake and growth retardation in broilers, the disease can lead to egg production drops of up to 40% in laying flocks (1, 21). Furthermore, recovered birds often become chronic carriers, and subsequent environmental or infectious stressors can readily trigger recurrent outbreaks, substantially complicating long-term disease control efforts.

*A. paragallinarum* has a global distribution and is particularly prevalent in intensively managed poultry operations across Asia, the Americas, Europe, and Africa (6, 22, 23). In China, the first confirmed reports of serotypes A, C, and B date from 1986, 1993, and 2003, respectively (15). Epidemiological investigations have consistently identified serotypes A and C as the predominant circulating types, with serotype B detected only sporadically (3, 24). Surveys conducted from 2019–2020 confirmed the co-circulation of all three serotypes, with a notable predominance of serotype C during that period (4). The epidemiology of *A. paragallinarum* in China exhibits distinct winter–spring seasonality and higher prevalence in southern provinces compared to northern regions (25, 26). Collectively, these findings underscore the dynamic and evolving nature of *A. paragallinarum* epidemiology in China, emphasizing the critical need for continuous surveillance.

This multi-provincial survey provides updated data on the spatial and temporal patterns of *A. paragallinarum* circulation in China. IC occurred year-round but exhibited strong seasonality, with distinct peaks in winter and spring—consistent with prior observations that cool, humid conditions favor bacterial survival and transmission (25). Positive samples were mainly concentrated in Jiangsu, Guangxi, and Guangdong, while Sichuan, Zhejiang, and Hubei contributed only sporadic detections. The higher prevalence in southern coastal provinces likely reflects intensive poultry production, frequent bird movements, and climatic conditions that favor pathogen persistence. Serotype A was predominated in Jiangsu and Guangdong, while both serotypes A and C co-circulated in Guangxi. Although serotype B and serotype C were detected at low frequency, their continued presence suggests ongoing, albeit limited, transmission cycles. These epidemiological patterns reaffirm the necessity for region-specific surveillance programs and evidence-based vaccine selection.

A notable observation in this study was the discrepancy between the number of PCR-positive samples and the relatively small number of successfully isolated strains. This outcome is expected and reflects fundamental methodological differences between molecular detection and bacterial isolation. PCR assays detect bacterial DNA regardless of organism viability, whereas successful isolation of *A. paragallinarum* is strongly influenced by sample freshness, transport conditions, prior antimicrobial treatment, and the organism’s fastidious growth requirements. Similar discrepancies between PCR detection rates and isolation success have been reported previously (24). Importantly, this limitation does not undermine the epidemiological significance of PCR-based surveillance but highlights the need for cautious interpretation when correlating molecular positivity with culture recovery.

Our findings on pathogenicity and transmission dynamics align closely with previous studies. Zhao et al. reported that clinical signs appeared earlier following direct infraorbital sinus inoculation than after natural contact exposure, with peak signs in the latter group delayed by 3–5 days (26). This pattern is consistent with the 4-day delay we observed in contact-sentinel birds compared to directly inoculated chickens. Similarly, Guo et al. documented rapid development of inflammatory lesions in the sinus and tracheal mucosa as early as 3 days post-infection (24). Earlier research demonstrated that *A. paragallinarum* adheres tightly to ciliated epithelial surfaces, inducing epithelial desquamation and airway narrowing (24, 27).

The extensive tissue destruction observed in this study is likely attributable, in part, to toxin-mediated injury. Several studies have implicated RTX (Repeats-in-ToXin) family toxins in the pathogenesis of *A. paragallinarum* (27). These toxins form pores in host cell membranes, leading to osmotic lysis, ionic dysregulation, and necrotic cell death (28, 29). Putative RTX-encoding genes have been identified in the genome of *A. paragallinarum*, and RTX1-like proteins have been characterized in related Avibacterium species (28, 29). Furthermore, Gram-negative outer membrane vesicles (OMVs) contribute to virulence by facilitating biofilm formation, stimulating potent pro-inflammatory responses, and delivering cytotoxic cargo to host tissues (30). It is plausible that OMVs derived from *A. paragallinarum* function similarly *in vivo*.

Host species and age are critical determinants of *A. paragallinarum* pathogenicity. Although cross-species transmission can occur, field isolates demonstrate strong host preference, with chicken- and goose-origin strains exhibiting high virulence in their natural hosts but attenuated effects in heterologous species (23). Balouria et al. reported pronounced differences in clinical presentation between chickens and Japanese quail, further supporting this host-restricted virulence (23). Age-related susceptibility is another key factor. In our study, 50-day-old SPF chickens developed clinical signs at 24 hpc. In contrast, younger birds (2.5 weeks old) in Balouria’s work exhibited symptoms within 12 h (23), suggesting that juvenile birds may experience a more rapid and potentially more severe disease onset.

These observations reinforce that clinical outcome reflects the combined effects of strain virulence, host susceptibility, and environmental conditions. Efficient horizontal transmission observed for both serotypes further underscores the potential for rapid flock-to-flock spread under commercial conditions.

In conclusion, IC remains a significant challenge for Chinese poultry industry. *A. paragallinarum* is widely distributed, with serotypes A and C currently predominating and exhibiting high virulence and transmissibility. Although serotype B circulates sporadically, its persistence necessitates ongoing surveillance. Effective control of IC requires a multifaceted strategy combining regionally tailored vaccination—based on prevalent serotypes—with stringent biosecurity measures to interrupt transmission. Sustained epidemiological monitoring is essential to track serotype dynamics, inform vaccine updates, and enable the development of targeted, effective prevention strategies.

## Data Availability

The original contributions presented in the study are included in the article/supplementary material, further inquiries can be directed to the corresponding authors.
